# Metastatic melanoma: an unexpected cause of acute liver failure

**DOI:** 10.1007/s12328-024-02039-1

**Published:** 2024-09-17

**Authors:** Robert S. O’Neill, Phillip Leaver, Connor Ryan, Sharron Liang, Santosh Sanagapalli, Rasha Cosman

**Affiliations:** 1 Faculty of Medicine, Vincent’s Clinical School, University of New South Wales, Sydney, Australia; 2 Department of Gastroenterology and Hepatology, Vincent’s Hospital, Sydney, Australia; 3 Department of Anatomical Pathology, Sydpath, St Vincent’s Hospital, Sydney, NSW Australia; 4 Department of Medical Oncology, St Vincent’s Hospital, Sydney, NSW Australia; 5 The Kinghorn Cancer Centre, Sydney, NSW Australia; 6 Garvan Institute for Medical Research, Sydney, NSW Australia

**Keywords:** Melanoma, Hepatitis, Acute liver failure, Metastatic melanoma

## Abstract

Acute liver failure secondary to metastatic melanoma is exceedingly rare with the literature limited to case reports. The disease itself presents with vague symptoms making diagnosis difficult without a high clinical suspicion. Further to this, the prognosis of acute liver failure secondary to metastatic melanoma is dismal. We present the case of a 59-year-old male with a distant history of previously excised cutaneous melanoma who presented to our institution with abdominal pain and liver enzyme derangement suggestive of acute hepatitis. Due to progressive derangement in liver function and cross-sectional imaging suggestive of an infiltrative cause, a left axillary lymph node was biopsied which demonstrated metastatic melanoma. The patient subsequently deteriorated into acute liver failure and despite acute treatment of his underlying metastatic melanoma died 17 days post initial presentation. This case highlights an uncommon cause of acute liver failure as well as the poor prognosis associated with acute liver failure secondary to metastatic melanoma.

## Introduction

Melanoma is a disease of melanocytes with the vast majority of cases being of cutaneous in origin [1]. Malignant melanoma (MM) is an aggressive malignancy with an unpredictable metastatic pattern, with the most common organs involved being the lungs, bone, lymph nodes, liver and brain [2]. Liver metastasis are detected in approximately 10–20% of patients diagnosed with MM, however, liver dysfunction is exceedingly uncommon [3]. Survival rates vary between the subtypes of melanoma, with mucosal and uveal portending a poor prognosis, however, 5-year survival rates in those diagnosed with stage IV cutaneous melanoma has been previously documented at 34.3% [4]. Further to this, liver failure secondary to MM is rare with the literature limited to case reports and is associated with a dismal prognosis [5]. We present the case of a 59-year-old male who presented with hepatitis who subsequently progressed into acute hepatic failure secondary to metastatic melanoma of cutaneous origin.

## Case report

A 59-year-old male presented to our institution with a 7-day history of right upper quadrant pain, myalgia, nausea and subjective fevers. He had a history notable for cutaneous actinic keratosis and cutaneous melanoma in situ localised on the upper back, previously excised one year prior to presentation with clear surgical margins on histopathological assessment of the post-operative specimen. He was on no regular medications. Examination on admission demonstrated right upper quadrant and epigastric tenderness. Biochemical evaluation revealed liver enzyme derangement with an aspartate transferase (AST) of 353 U/L, alanine aminotransferase (ALT) of 616 U/L, alkaline phosphatase (ALP) of 329 U/L and gamma-glutamyl transferase (GGT) of 507 U/L. Bilirubin was elevated to 23 mmol/L. Lactate dehydrogenase (LDH) and ferritin were elevated to 1640 U/L and 2350 ug/L, respectively. His INR was 1.4 and prothrombin time was elevated to 19 s. The remainder of his biochemical assessment for acute liver dysfunction was normal.

**Fig. 1:**
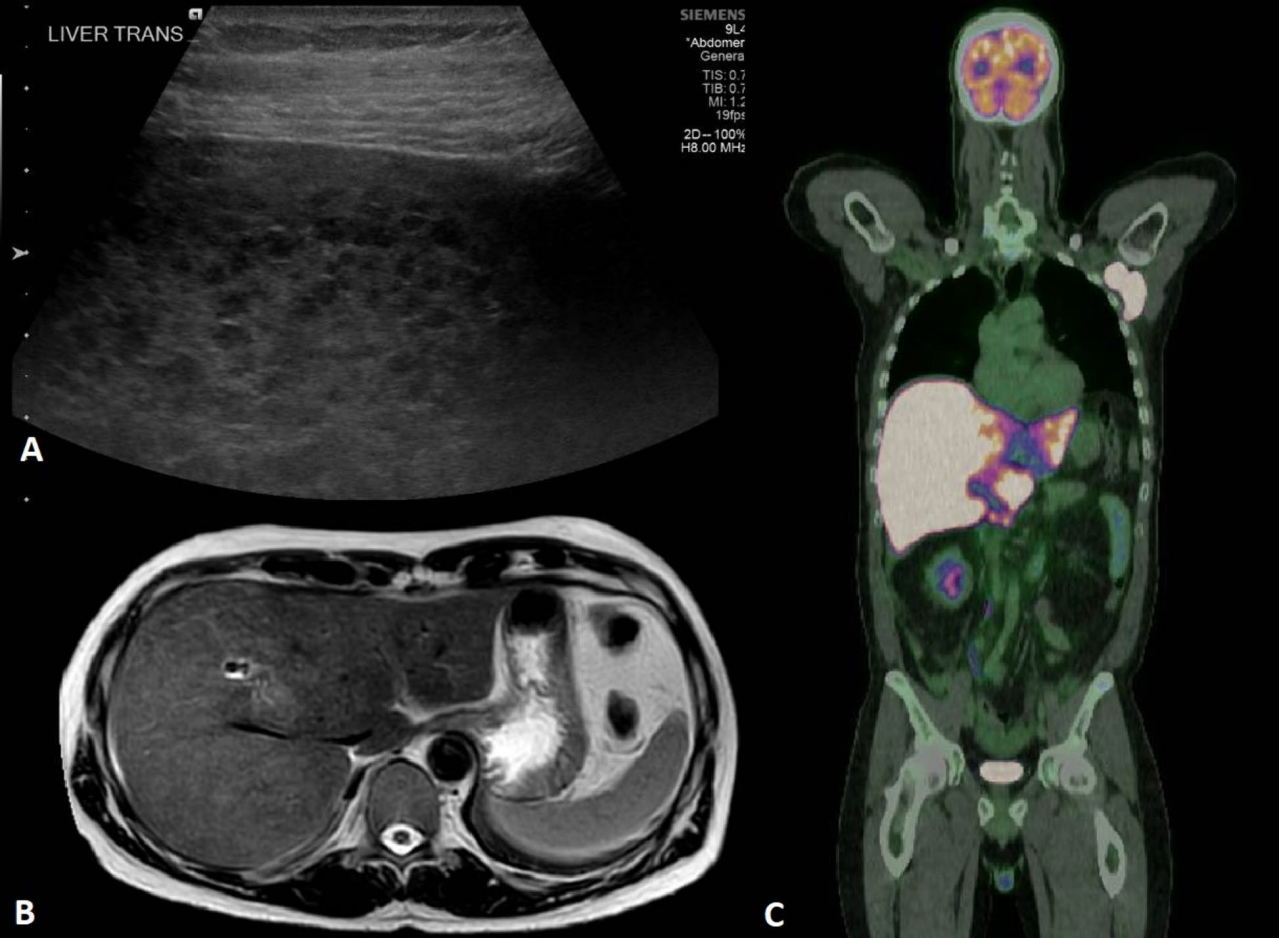
**A** Abdominal ultrasound demonstrating hepatic nodularity; **B** MRCP demonstrating a diffusely abnormal and heterogenous liver; **C** FDG-PET/CT scan revealed intensely FDG avid left axillary, peripancreatic and portocaval lymphadenopathy

Abdominal ultrasound demonstrated a coarse liver echotexture with nodularity along with splenic nodularity (Fig. 1A). Subsequent magnetic resonance cholangiopancreatography (MRCP) revealed a diffusely abnormal and heterogenous liver with five focal lesions along with multiple thoracic and lumbar bone lesions (Fig. 1B). Inpatient FDG-PET/CT scan revealed intensely FDG avid left axillary, peripancreatic and portocaval lymphadenopathy (Fig. 1C). Further to this, splenic nodules along with numerous FDG avid bone lesions were identified. His liver demonstrated global diffusely increased uptake along with superimposed intense foci throughout.

**Fig. 2:**
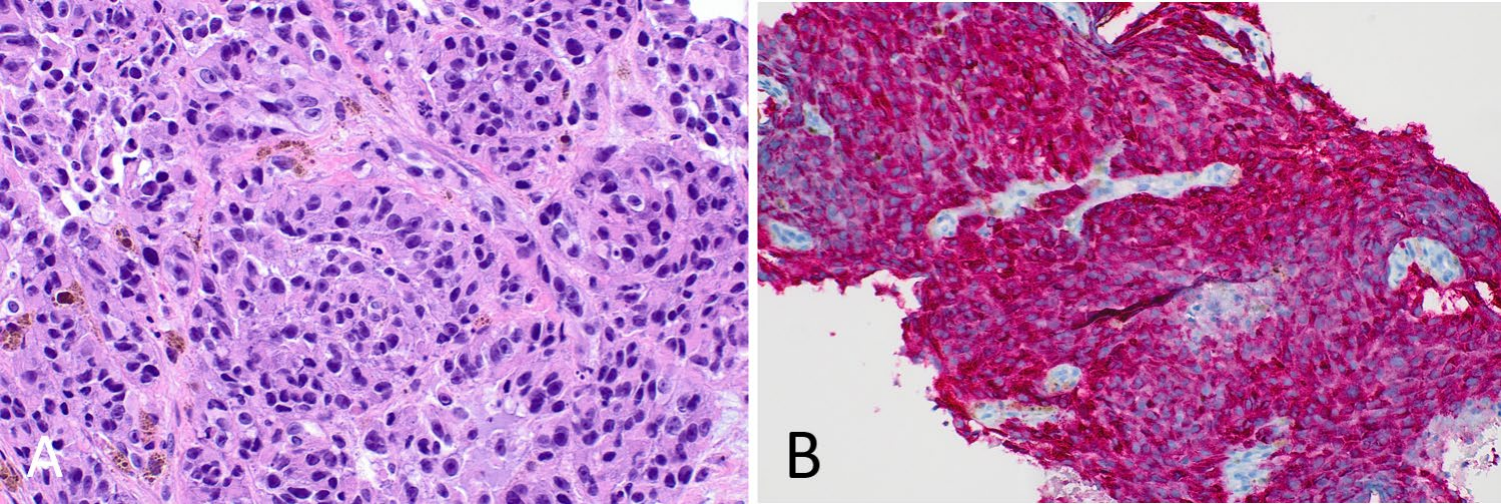
**A** Hematoxylin and eosin staining of left axillary lymph node core biopsy demonstrating discohesive nests of epithelioid cells showing hyperchromatic nuclei and nucleoli. Prominent pigment is present. **B** Immunohistochemistry (IHC) for MART1 is positive consistent with metastatic melanoma

On day 5 of the patient’s admission, core biopsy was performed of the left axillary lymph node. Histopathological analysis of the left axillary lymph node core biopsy revealed discohesive nests of epithelioid cells showing enlarged, hyperchromatic nuclei, nucleoli and many containing pigment on hematoxylin and eosin staining (Fig. 1A). Subsequent immunohistochemistry demonstrated positive staining for MART1 (Fig. 1B), along with SOX10 and HMB45. A diagnosis of metastatic melanoma was made with an acute liver injury secondary to hepatic infiltration. Targeted mutational analysis using next generation sequencing identified a NRAS Q61L gene mutation (Fig. 2).

**Fig. 3:**
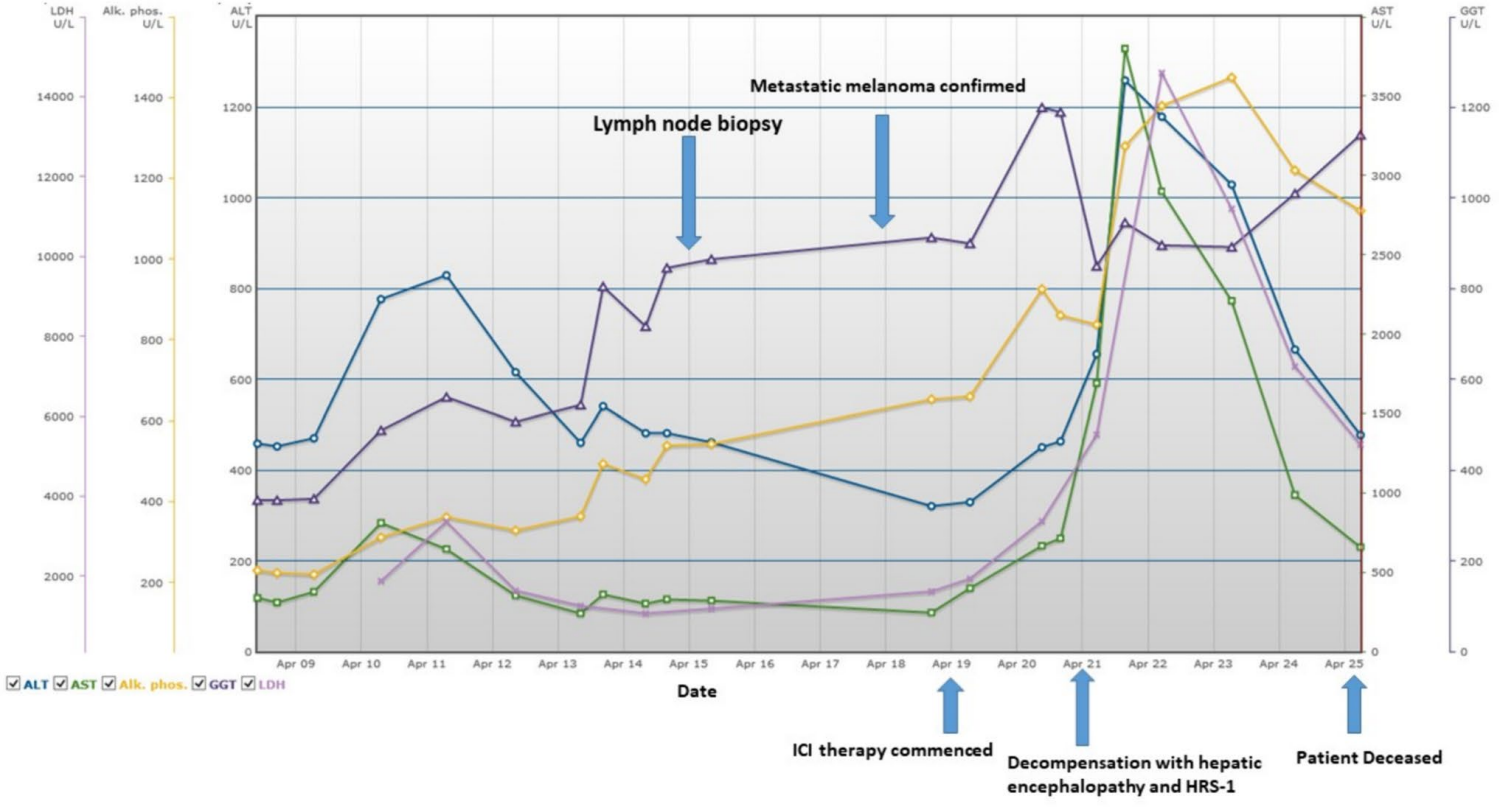
Line graph depicting clinical and biochemical deterioration during inpatient admission. *ICI* – immune checkpoint inhibitor; *HRS-1* – hepatorenal syndrome type 1. *AST* – aspartate aminotransferase; *ALT* – alanine aminotransferase; *Alk*. *Phos*.—alkaline phosphatase; *GGT*—Gamma Glutamyl Transferase; *LDH* – Lactate dehydrogenase

The patient was commenced on dual immunotherapy with nivolumab and ipilimumab. Two days after immunotherapy commencement the patient deteriorated with decompensated liver failure secondary to hepatic infiltration, with the development of hepatorenal syndrome and ascites. His AST and ALT increased to 2900 IU/L and 1180 IU/L, respectively. Serum bilirubin was also significantly elevated to 100 mmol/L, along with LDH to 14,600 U/L (Fig. 3). Despite treatment with intravenous terlipressin and intensive care admission, the patient deteriorated with refractory encephalopathy and type one hepatorenal syndrome. A palliative approach was adopted. The patient died 17 days post initial presentation.

## Discussion

Melanoma, although only making up a small subset of cutaneous malignancies, accounts for a significant amount of morbidity and mortality. This is reportedly secondary to the high metastatic propensity of the tumour [6]. Liver metastasis secondary to malignant melanoma is relatively common, however, substantial replacement of liver by tumour resulting in impaired liver function is rare and limited to case reports [3, 7–20]. This case shares similarities with previously published cases, namely the rapid progression to liver failure and subsequent death,

Histologically, melanoma dissemination is characterized by leukemoid tumour infiltration of the liver sinusoids leading to obstruction, hepatocellular death, and liver failure [19]. Clinically, hepatic infiltration of MM is difficult to distinguish from other primary causes of hepatitis. Biochemical signs that increase suspicion of an underlying malignant aetiology include mild to moderately elevated serum aminotransferases, ALP and bilirubin levels, while an elevated lactate dehydrogenase (LDH) has been associated with tumour involvement previously [5]. LDH elevation has been previously identified as an ominous sign in the context of acute liver failure secondary to metastatic malignancy, as demonstrated in the presented case [8, 11, 17]. This is perceived to be secondary to extensive cellular ischaemia and necrosis, or related to release of LDH from the tumor cells themselves [8]. Imaging features are usually non-specific, however, hepatomegaly without defined nodular lesions is a usual manifestation of diffuse hepatic melanoma infiltration [20].

Previously, the pathophysiology of acute liver failure in the context of MM was postulated to be secondary to hepatic ischemia arising from sinusoidal infiltration and vessel invasion. This is supported in the case of the presented patient by the dramatic rise in serum aminotransferases and LDH, as well as the presence of pain [11]. It has also been hypothesised that simple replacement of the liver parenchyma by melanoma itself results in rapid liver failure [8].

Clinically, patients present with non-specific symptoms such as pain, fatigue, nausea and anorexia. The prognosis of the disease is exceedingly poor with life expectancy ranging from days to months based on previous case reports, while the diagnosis is commonly made posthumously due to severe coagulopathy precluding liver biopsy [20]. Treatment focuses on targeting the underling malignancy with a view to stabilise hepatic function to prevent further decompensation, however, given the degree of liver involvement in the presented case, it was deemed that despite immunotherapy, rapid progression of his underlying acute hepatic failure was the likely cause of death.

This case highlights an exceedingly uncommon cause of acute hepatic failure which initially presented with features suggestive of hepatitis. A potential diagnosis of immune checkpoint inhibitor hepatitis was flagged as a differential diagnosis, however, due to the coagulopathy, a biopsy was not pursued. MM is an extremely rare cause of acute liver failure with the literature limited to case reports. In such cases, the prognosis is poor, as demonstrated with the presented patient. Future research should aim to focus on the identification of early non-invasive biomarkers that could assist in aiding clinicians in the early diagnosis of hepatic metastasis in the case of metastatic melanoma. Early identification of hepatic disease in metastatic melanoma should prompt clinicians to implement or escalate therapy given the poor prognosis associated with progression to hepatic failure. In the case of rapid liver failure secondary to MM, the pathophysiology of the disease process is a relatively unknown entity given the lack of published literature pertaining to it. With earlier identification of metastatic disease, perhaps future studies could aim to focus on the tumor microenvironment in the liver to determine the processes that occur at a cellular level that drives progression to liver failure. In the case of abdominal pain with progressive liver function derangement and subsequent acute hepatic failure, metastatic malignancy, including but not limited to MM, should be considered as a potential diagnosis given its associated poor prognosis.

## References

[1] Curti BD, Leachman S, Urba WJ. Cancer of the Skin. In: Jameson JL, Fauci AS, Kasper DL, Hauser SL, Longo DL, Loscalzo J, editors. Harrison’s principles of internal medicine. 20th ed. New York (NY): McGraw-Hill Education; 2018. p. 522–30.

[2] Chang AE, Karnell LH, Menck HR. The National Cancer Data Base report on cutaneous and noncutaneous melanoma: a summary of 84,836 cases from the past decade. The American College of Surgeons Commission on Cancer and the American Cancer Society. Cancer. 1998;83:1664–78. 9781962 10.1002/(sici)1097-0142(19981015)83:8<1664::aid-cncr23>3.0.co;2-g

[3] Rose DM, Essner R, Hughes TM, et al. Surgical resection for metastatic melanoma to the liver: the John Wayne Cancer Institute and Sydney Melanoma Unit experience. Arch Surg. 2001;136:950–5. 11485537 10.1001/archsurg.136.8.950

[4] SEER S. Explorer: An Interactive Website for SEER Cancer Statistics. Surveillance Research Program, National Cancer Institute. Available online: Accessed 27 Sept 2021

[5] Lee Y, Lee J, Kim H, et al. Acute liver failure secondary to hepatic infiltration of malignant melanoma. Clinical Endoscopy. 2021;55:287–91. 33789416 10.5946/ce.2020.272 PMC8995978

[6] Damsky WE Jr, Rosenbaum LE, Bosenberg M. Decoding melanoma metastasis. Cancers. 2010;3:126–63. 24212610 10.3390/cancers3010126 PMC3756353

[7] Fusasaki T, Narita R, Hiura M, et al. Acute hepatic failure secondary to extensive hepatic replacement by metastatic amelanotic melanoma: an autopsy case report. Clin J Gastroenterol. 2010;3:327–31. 26190491 10.1007/s12328-010-0181-x

[8] Te HS, Schiano TD, Kahaleh M, et al. Fulminant hepatic failure secondary to malignant melanoma: case report and review of the literature. Am J Gastroenterol. 1999;94:262–6. 9934768 10.1111/j.1572-0241.1999.00811.x

[9] Montero JL, Muntané J, de las Heras S, et al. Acute liver failure caused by diffuse hepatic melanoma infiltration. J Hepatol. 2002;37(4):540–1. 12217611 10.1016/s0168-8278(02)00219-2

[10] Velázquez HE, Castro-Alonso FJ, Bourlon C, et al. Diffuse hepatic infiltration by metastatic melanoma. Oncology. 2019;33:629386. 31365754

[11] Schlevogt B, Rehkämper J, Hild B, et al. Hepatobiliary and Pancreatic: Fulminant liver failure from diffuse leukemoid hepatic infiltration of melanoma. J Gastroenterol Hepatol. 2017;32(11):1795. 29024015 10.1111/jgh.13904

[12] Tanaka K, Tomita H, Hisamatsu K, et al. Acute liver failure associated with diffuse hepatic infiltration of malignant melanoma of unknown primary origin. Intern Med. 2015;54:1361–4. 26027987 10.2169/internalmedicine.54.3428

[13] Bellolio E, Schafer F, Becker R, et al. Fulminant hepatic failure secondary to diffuse melanoma infiltration in a patient with a breast cancer history. J Postgrad Med. 2013;59:164–6. 23793331 10.4103/0022-3859.113822

[14] Mashayekhi S, Gharaie S, Hajhosseiny R, et al. A rare presentation of malignant melanoma with acute hepatic and consecutive multisystem organ failure. Int J Dermatol. 2014. 10.1111/ijd.12268. 24168059 10.1111/ijd.12268

[15] Shan GD, Xu GQ, Chen LH, et al. Diffuse liver infiltration by melanoma of unknown primary origin: one case report and literature review. Intern Med. 2009;48:2093–6. 20009398 10.2169/internalmedicine.48.2542

[16] Rubio S, Barbero-Villares A, Reina T, et al. Rapidly-progressive liver failure secondary to melanoma infiltration. Gastroenterol Hepatol. 2005;28:619–21. 16373011 10.1016/s0210-5705(05)71525-9

[17] Pichon N, Delage-Corre M, Paraf F. Hepatic metastatic miliaria from a malignant melanoma: 2 case reports. Gastroenterol Clin Biol. 2004;28:593–5. 15243393 10.1016/s0399-8320(04)95016-6

[18] Bouloux PM, Scott RJ, Goligher JE, et al. Fulminant hepatic failure secondary to diffuse liver infiltration by melanoma. J R Soc Med. 1986;79:302–3. 3723524 10.1177/014107688607900513 PMC1290318

[19] Kaplan GG, Medlicott S, Culleton B, et al. Acute hepatic failure and multi-system organ failure secondary to replacement of the liver with metastatic melanoma. BMC Cancer. 2005;5:1–3. 15989692 10.1186/1471-2407-5-67 PMC1192792

[20] Aierken Y, Zhu YF, Yang JY. Acute liver failure caused by metastatic malignant melanoma of liver: A case report. Medicine: Case Reports and Study Protocols; 2021. 10.1097/MD9.0000000000000080.

